# Impacts of Motion-Based Technology on Balance, Movement Confidence, and Cognitive Function Among People With Dementia or Mild Cognitive Impairment: Protocol for a Quasi-Experimental Pre- and Posttest Study

**DOI:** 10.2196/18209

**Published:** 2020-09-18

**Authors:** Erica Dove, Rosalie Wang, Karl Zabjek, Arlene Astell

**Affiliations:** 1 Rehabilitation Sciences Institute University of Toronto Toronto, ON Canada; 2 Toronto Rehabilitation Institute University Health Network Toronto, ON Canada

**Keywords:** motion-based technology, dementia, mild cognitive impairment, cognitive dysfunction, postural balance, movement confidence, cognition, exercise movement techniques

## Abstract

**Background:**

While exercise can benefit the health and well-being of people with dementia or mild cognitive impairment, many exercise programs offered to this population are passive, unengaging, and inaccessible, resulting in poor adherence. Motion-based technologies are increasingly being explored to encourage exercise participation among people with dementia or mild cognitive impairment. However, the impacts of using motion-based technologies with people with dementia or mild cognitive impairment on variables including balance, movement confidence, and cognitive function have yet to be determined.

**Objective:**

The purpose of this study is to examine the impacts of a group motion-based technology intervention on balance, movement confidence, and cognitive function among people with dementia or mild cognitive impairment.

**Methods:**

In this quasi-experimental pre- and posttest design, we will recruit 24 people with dementia or mild cognitive impairment from 4 adult day programs and invite them to play Xbox Kinect bowling in a group setting, twice weekly for 10 weeks. We will require participants to speak and understand English, be without visual impairment, and be able to stand and walk. At pretest, participants will complete the Mini-Balance Evaluation Systems Test (Mini-BESTest) and the Montreal Cognitive Assessment (MoCA). We will video record participants during weeks 1, 5, and 10 of the intervention to capture behavioral indicators of movement confidence (eg, fluency of motion) through coding. At posttest, the Mini-BESTest and MoCA will be repeated. We will analyze quantitative data collected through the Mini-BESTest and the MoCA using an intent-to-treat analysis, with study site and number of intervention sessions attended as covariates. To analyze the videos, we will extract count and percentage data from the coded recordings.

**Results:**

This study will address the question of whether a group motion-based technology intervention, delivered in an adult day program context, has the potential to impact balance, movement confidence, and cognitive function among people with dementia or mild cognitive impairment. The project was funded in 2019 and enrollment was completed on February 28, 2020. Data analysis is underway and the first results are expected to be submitted for publication in 2021.

**Conclusions:**

This study will assess the feasibility and potential benefits of using motion-based technology to deliver exercise interventions to people with dementia or mild cognitive impairment. This work can also be used as the basis for developing specific software and future exercise programs using motion-based technology for people with dementia or mild cognitive impairment, as well as understanding some of the conditions in which these programs can be delivered.

**International Registered Report Identifier (IRRID):**

DERR1-10.2196/18209

## Introduction

### Background

Major neurocognitive disorder, also known as *dementia*, is defined as a significant decline of 2 or more standard deviations from a previous level of performance in 1 or more cognitive domains: executive function, learning and memory, complex attention, language, perceptual-motor abilities, and social cognition [1]. Causes of dementia include Alzheimer disease, cerebrovascular disease, and other conditions that affect the brain [2]. The estimated number of people living with dementia worldwide is 50 million [3], which is predicted to reach 152 million by 2050 [4].

Mild neurocognitive disorder, also known as *mild cognitive impairment* (MCI), is a common precursor to dementia, although not all people with MCI progress to dementia. MCI is characterized as cognitive decline greater than would be expected for a person’s age in 1 cognitive domain that does not interfere with daily activities (eg, leisure) [1]. While MCI is not necessarily progressive, it is estimated that roughly 8.7% of people with MCI progress to dementia each year [5]. There are no pharmacological interventions to reverse cognitive changes or maintain the cognitive functioning of people with dementia or MCI [6]. As such there is an urgent need for effective interventions that support people to live well with dementia or MCI. This includes addressing physical challenges such as falls, which can undermine independence and increase the risk of hospitalization or transfer to long-term care.

People with dementia experience 2 to 8 times more falls than older people without dementia [7]. This has variously been attributed to balance impairments [8], a lack of movement confidence [9], and poor cognitive function or severity of cognitive impairment [10]. Taking balance first, people with dementia or MCI have an increased risk of developing balance impairments compared with older adults without cognitive impairment [11]. Indeed, a study involving people with subjective cognitive impairment, MCI, and Alzheimer disease found that all aspects of balance deteriorated with increasing severity of cognitive impairment [12].

Movement confidence is defined as “a person’s feeling or sense of adequacy in a movement situation” [13] (pg 213). Movement confidence relies heavily on an individual’s implicit belief or perception that they have the skills necessary to successfully perform a movement task [13,14]. Individuals who are movement confident are more likely to participate in movement activities to their satisfaction, whereas nonconfident individuals are less likely to participate in movement activities or find it less satisfying to participate [13,15]. This is important given that an individual’s level of movement confidence relates directly to their engagement with and level of participation in physical activity and exercise [16]. Lack of movement confidence is associated with an increased risk of falling among older adults, in general [17]. Among people with dementia or MCI, lack of movement confidence is prevalent [18,19], although no studies have examined the direct link between lack of movement confidence and risk of falling. Given the above, there is a need for practical interventions that mitigate impairments in movement confidence among people with dementia or MCI. Such interventions could include physical activity and exercise, as these interventions might also impact other areas of physical function, to ensure that older people and people with dementia or MCI have the physical capacity to support the undertaking of higher-risk movements.

Exercise, independent of type (eg, cardiovascular, strength, balance and flexibility), offers a range of benefits, including decreased risk of falls, reduced risk of chronic disease, increased physical function (eg, balance), and improved cognitive function [20]. According to the Ontario Brain Institute [21], people with dementia should follow the same exercise guidelines provided by the Canadian Society for Exercise Physiology for older adults (ie, people aged ≥65 years) [22]. These guidelines recommend participation in roughly 150 minutes of moderate to vigorous physical activity per week, with moderate to vigorous physical activity deemed as activities that produce slight perspiration and a slight increase in breathing rate. It is recommended that for older adults and people with dementia moderate to vigorous physical activity consist of enjoyable activities that include cardiovascular (eg, brisk walking), strength training (eg, lifting weights), and balance exercises (eg, tai chi) [22]. The MCI practice guidelines [23] suggest that exercising twice weekly has benefits for cognition and general health. However, they do not mention the specific duration (ie, number of minutes) or type(s) of exercise to prescribe to people with MCI for optimal health benefits. However, despite the evident benefits of exercise for older people and people with dementia or MCI, only 37.3% of people aged 65 and over in Canada meet the recommended guidelines for exercise participation [24], with these numbers decreasing with advanced age and with the presence of chronic diseases, such as dementia [25]. Technology-based gaming or “exergaming” is emerging as a promising approach to overcome barriers and increase exercise participation by older adults and those with additional needs [26].

Motion-based technology (a type of exergaming technology) operates through human gestures [27]. In motion-based games, actions made in the real world (eg, reaching) are replicated by a virtual character on a screen (eg, a game character grabbing a ball). Motion-based technology has recently grown in popularity as a tool in rehabilitation and scientific research [28] with older adults [29], as well as people with Parkinson disease [30], multiple sclerosis [31], traumatic brain injury [32], and stroke [33]. For example, Pompeu and colleagues [30] showed improvements in balance among people with Parkinson disease after taking part in a 10-week motion-based technology program of 45- to 60-minute sessions 3 days per week involving a series of Nintendo Wii balance games.

A review of the use of motion-based technologies by people with dementia or MCI demonstrated that these technologies can provide enjoyable cognitive, physical, and leisure activities [27]. From a rehabilitation perspective, Schwenk and colleagues [34] showed improvements in balance and fear of falling among people with dementia or MCI after participating in a 4-week virtual reality motion-based technology program held twice weekly. Similarly, Amjad and colleagues [35] showed significant improvements in cognitive function in their participants with dementia or MCI after taking part in a 6-week motion-based technology program, held 5 times per week for 25 to 30 minutes. Moreover, when motion-based technologies are used in a group setting, they allow participants to socialize with and support one another. For example, people with dementia or MCI can play Xbox Kinect bowling “independently together” through cheering, laughing, and friendly competition [36]. Some motion-based systems have additional advantages in terms of accessibility and inclusivity, such as accommodating players who use mobility devices such as walkers and wheelchairs [37].

### Objective

Despite these promising results, the impacts of interventions using motion-based technology are relatively unknown for this population. In their review of the efficacy of motion-based technology interventions for people with dementia with regard to cognition, physical function (eg, strength), emotional well-being, social health, and quality of life, van Santen and colleagues [38] identified only 3 randomized trials. We designed this study to extend knowledge of the potential of motion-based technology in rehabilitation for people with dementia or MCI. Specifically, we will examine the impacts of a group motion-based technology intervention on balance, movement confidence, and cognitive function among people with dementia or MCI. The specific hypotheses of the study reflect these aims. Hypothesis 1 states that balance will show statistically significant improvement after the motion-based technology intervention, as measured by the Mini-Balance Evaluation Systems Test (Mini-BESTest). Hypothesis 2 states that movement confidence will improve after the motion-based technology intervention, as measured by the analysis of coded video-recorded footage taken of participants during the intervention sessions (eg, number of turns completed confidently). Hypothesis 3 states that cognitive function will show statistically significant differences after the motion-based technology intervention, as measured by the Montreal Cognitive Assessment (MoCA).

## Methods

### Study Design

This study will use a standard within-participants design, with measurement at pre- and postintervention (1 group, 2 times).

### Ethics

We received ethical approval to conduct this study from the Health Sciences Research Ethics Board at the University of Toronto (#37326) and from the University Health Network Research Ethics Board (#19-5524), Toronto, ON, Canada. Participants will be able to withdraw their consent to participate in the study at any time by informing the research team in person or using the contact information provided in the informed consent forms. We will not compensate participants for their participation in the study given that they will already be at the day program when the intervention is scheduled to take place, meaning that we do not expect travel-related costs associated with the study. If a participant injures themselves at any point during their participation in the study, we will seek medical attention immediately.

### Participants

We will recruit a convenience sample of 24 people with dementia or MCI from 4 community-based adult day programs. The sample size calculation was based on detecting the minimum clinical important difference of 4.0 points [39] on the primary outcome measure (Mini-BESTest) [40], using an effect size of 0.8, an alpha value of .05, a power value of 80%, and a 2-tailed *t* test. We calculated the estimated sample size to be at least 15 participants, which we increased to 24 participants to allow for 40% attrition. We chose an attrition rate of 40% given that absenteeism and unforeseen events (eg, prolonged illness, moving to long-term care) are common among adult day program clients.

Prospective participants will be required to (1) attend the day program at least twice per week on the days in which the intervention is to be offered; (2) meet the screening criteria for the presence of cognitive impairment (determined by a score of ≤25 on the MoCA); (3) speak and understand English well enough to recognize instructions from the facilitator and respond accurately; (4) not have visual impairment that would prevent the participant to view the game screen; (5) be able to stand and walk, with or without an assistive device (eg, walker), to complete the Mini-BESTest; and (6) have the capacity to provide informed consent, or have a substitute decision maker who can provide informed consent. We will exclude participants who do not meet the above criteria from participating in the study.

### Materials

#### Eligibility Screening Questionnaire

We will use an eligibility screening questionnaire, which we developed, to objectively record whether participants meet each item listed in the inclusion criteria. Screening questions pertain to day program schedule attendance, presence or absence of visual impairment, mobility status, presence or absence of cognitive impairment, ability to speak and understand English, and capacity to provide informed consent.

#### Capacity Recording Tool

We will use a capacity recording tool, along with probing questions from a pocket guide for determining a participant’s decision-making capacity [41]. We will use these questions while reviewing the informed consent form to determine whether the prospective participant meets the following criteria: (1) ability to understand relevant information; (2) ability to appreciate the situation and its consequences; (3) ability to reason; and (4) ability to communicate and express a choice. We will determine the participants who meet the 4 criteria to have the capacity to provide informed consent at that time.

#### Demographic Questionnaire

We will use a demographic questionnaire, which we developed, to capture participants’ descriptive information, including age, sex, education level, mobility device use, current exercise participation (ie, how many days per week they engage in exercise, as well as the types of exercise they engage in), current and past (ie, within 12 months) rehabilitation service use for balance impairments (eg, physiotherapy), previous bowling experience, and previous experience using motion-based technologies.

#### MoCA

The MoCA is a brief assessment of global cognitive function [42]. The MoCA assesses individual domains of cognitive function, including attention and concentration, executive functions, memory, language, visuoconstructional skills, conceptual thinking, calculations, and orientation. The MoCA results in scores for the individual cognitive domains that contribute to an overall total. The MoCA is scored out of 30 points, with a score of 25 points or less indicating the presence of cognitive impairment [42]. More specifically, a score of below 23 points on the MoCA indicates MCI [43] and a score of below 17 points indicates Alzheimer disease [44].

#### Mini-BESTest

The Mini-BESTest [40] is a 14-item comprehensive measure that simultaneously assesses several components of balance: anticipatory movements, reactive postural control, sensory orientation, and dynamic gait [45]. To complete the Mini-BESTest, participants are required to perform low-risk activities such as sit to stand, walking at different speeds, standing on a firm surface, and standing on 1 leg. The Mini-BESTest is scored out of 28 points, with higher scores indicating lesser impairments in balance [40]. While there are no prior studies using the Mini-BESTest with people with dementia or MCI, the Mini-BESTest has been shown to have excellent reliability (test-retest and interrater), internal consistency, sensitivity, responsiveness, and validity with populations similar to people with dementia or MCI, such as older adults [46], people with Parkinson disease [47], people with balance disorders [39], and people with chronic stroke [48].

### Intervention Description

We will invite participants to take part in a 10-week motion-based technology intervention (Xbox Kinect bowling), held in a group setting at 4 community-based adult day programs. The intervention sessions will take place twice per week for 10 weeks (20 sessions per site). Sessions will be facilitated by the first author (ED) using teaching techniques successfully employed in previous studies involving motion-based technology and people with dementia or MCI, such as verbal prompts, gesture demonstrations, and physical assistance [37]. During each session, approximately six participants will be seated together in a room and will each have a turn to engage with the technology and game. The facilitator will call upon each participant in the group 1 at a time to take their turn, which will continue for the duration of each session (approximately 60 minutes). While the active player is engaging with the technology, the rest of the group will sit and observe.

The motion-based technology system to be used in this study is the Xbox One Kinect (Microsoft Corporation). We chose the Xbox One Kinect over other commercially available motion-based technology systems (eg, Nintendo Wii) given that interaction with this system requires no handheld controller and relies purely on naturalistic movements (eg, waving an arm). We will used the commercially available bowling game offered through the Kinect Sports Rivals package in the study, alongside the Xbox One Kinect. We chose the Kinect Sports Rivals bowling game given that the game of bowling is generally familiar.

To partake in the intervention, participants are required to rise from their chair, walk to the line (ie, a piece of black electrical tape placed on the floor in front of the technology to cue participants regarding where to stand), play the bowling game, walk back to the chair, and sit down. We will break down the movements required to play the bowling game for participants in a stepwise manner: (1) raise an arm above the head to activate the Kinect sensor; (2) reach the same arm out to the side; (3) close the hand of the extended arm to pick up a bowling ball; (4) extend the same arm back behind to wind up; (5) open the hand of the same arm to release the ball; and (6) swing the same arm forward to throw the ball.

### Environment

We will hold the intervention sessions in the activity room of each adult day program site, which includes a large television set or smartboard, with enough space for participants to sit between turns and to play the game. For consistency, we will configure each day program activity room identically at each site for all intervention sessions, including the placement of the technology, the chairs, the facilitator, and the participants. The facilitator of the intervention sessions will be situated next to the game screen during all sessions to support participants as required. On the days in which video recording occurs, the video cameras will be consistently setup at the front and back of the room during the intervention to capture a comprehensive view of the active players during each of their respective turns.

### Procedure

We will first ask potential participants to complete the eligibility screening questionnaire and the MoCA [42] to ensure that the study inclusion criteria are met. The MoCA will be conducted in person, in a quiet and private setting (ie, in a separate room) at each day program. The first author (ED) will be responsible for administering and scoring the MoCA. At this time, prospective participants will also rereview the informed consent with the researcher, who will then determine their capacity to provide informed consent using the decision-making ability and capacity-probing questions. Once a participant has reviewed the informed consent form to their satisfaction, they will be asked to sign the informed consent form in the presence of the researcher, provided that they have the capacity to independently provide informed consent. Given that decisions are time specific, we will review each participants’ capacity to consent on an ongoing basis (ie, at each point of interaction). All consent and assent forms will be signed in a quiet and private area at the day program.

Upon providing informed consent (whether through independent consent or substitute decision-maker consent plus participant assent), each participant will complete the demographic questionnaire and the Mini-BESTest [40]. We will complete the pretest measurements as close to the eligibility measurements and to the intervention as possible (ie, within 1-3 days) to ensure that the MoCA (used for eligibility and outcome purposes) and Mini-BESTest scores do not become outdated. We will conduct the Mini-BESTest in an open area (eg, exercise room) at each day program to ensure that there is enough space to perform the test. Additionally, 2 members of the research team will be present during the Mini-BESTest to ensure that participants can safely perform the test. That is, 1 research team member will evaluate the participants’ performance while the other member acts as a spotter.

**Figure 1:**
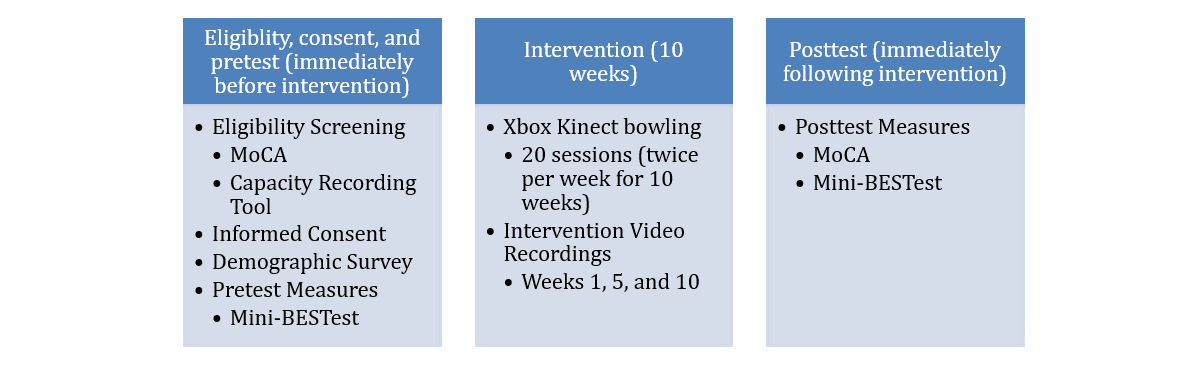
Study procedure. Mini-BESTest: Mini-Balance Evaluation Systems Test; MoCA: Montreal Cognitive Assessment.

Once the pretest measures are complete, participants will take part in a 10-week motion-based technology intervention (Xbox Kinect bowling), which will be held in a group setting at each day program. Sessions will be held twice per week for 10 weeks (20 sessions), with each session lasting approximately one hour. At the start of each session, the researcher will provide all participants with a verbal and physical demonstration of how to play the game. Additionally, we will record participant attendance to track how many sessions each participant takes part in over the 10-week intervention period. During weeks 1, 5, and 10 of the 10-week intervention period, we will take video recordings of each participant during the intervention using 2 video cameras to capture physical motions related to movement confidence. Immediately following the completion of the 10-week intervention (ie, within 1-3 days), all participants will complete the posttest measures, that is, the Mini-BESTest and the MoCA, for later comparison (Figure 1).

### Data Analysis

#### Inclusion in Data Analysis

We will amalgamate data collected from all 4 adult day programs for analysis and reporting purposes. We will encourage participants to attend as many of the intervention sessions as possible; using an intent-to-treat approach, we will include all participants in the final analysis (with number of sessions as a covariate), regardless of the number of intervention sessions they attended. Participants who miss sessions will still be permitted to participate in the motion-based technology intervention. Makeup sessions for participants who are not able to attend all sessions will not be offered. Participants who miss sessions will be accounted for during the analysis.

#### Analysis of Test and Questionnaire Data

All analyses will follow an intent-to-treat approach, whereby we will base the results of the experiment on all participants recruited to take part in the intervention, and not just those who complete the intervention [49]. This approach to analysis will provide a more reliable estimate of the true effectiveness of the motion-based technology intervention, by reproducing what occurs in real-world settings such as community-based adult day programs. We will handle missing data using multiple imputation, which is the recommended standard for accounting for missing research data when working with people who have dementia [50]. We will include study site and the number of intervention sessions attended by the participants in the statistical model as covariates. These analyses will be conducted using IBM SPSS version 26 (IBM Corporation), using a *P* value of <.05 and a confidence interval of 95%.

#### Analysis of Video-Recorded Data

We will code video-recorded data using behavioral analysis software (Observer XT version 12.0; Noldus Information Technology) [51] to capture potential indicators of movement confidence (eg, hesitation) and how these change over time. Behavioral analysis of video-recorded data involves creating a coding scheme with operationalized definitions to objectively code the data. Coded data can capture the frequency and duration of events and behaviors, such as how often confident or nonconfident behaviors occur. This will allow for the extraction of count and percentage data related to movement confidence (eg, 20% of turns were completed confidently during week 1, which increased to 80% by week 10).

We will develop the movement confidence coding scheme using information from 2 distinct sources: (1) previous studies that examined movement confidence (eg, [13,14,16]), and (2) observations from previous work with motion-based technology and people with dementia or MCI (eg, [36,37]). For instance, Dove and Astell [36] noted that people with dementia or MCI from an adult day program appeared to move more confidently with repeated exposure to the movement situation (ie, playing Xbox 360 Kinect bowling). That is, participants gradually transitioned from hesitant, rigid movements to relaxed, flowing movements and relied less on the instructor to complete the movement task. Indeed, over time, participants required fewer prompts (eg, verbal, gesture) and less physical support from the instructor to complete the movement task [36]. Additionally, some participants became less reliant on their mobility devices and began to develop a professional-looking bowling stance.

Video recordings will be coded by 4 independent raters, 1 of whom is the first author (ED). Prior to formally coding the videos for analysis, each rater will undergo training using the Observer XT video analysis software [51] and the movement confidence coding scheme. This will involve completing an introductory coding exercise, reviewing the video-recorded data, familiarizing themselves with the coding scheme and its operational definitions, and practicing coding the videos. We will compare the practice work of each coder for consistency using interrater reliability analysis to determine whether the 4 raters are reliably evaluating the same material [52]. This will also reduce the likelihood of researcher bias, given that the work of the other 3 raters will be compared for consistency against that of the fourth rater (ED), who is also involved in facilitating the study intervention and conducting the pretest and posttest measures. Once interrater reliability between the 4 raters reaches at least 80% agreement, the 4 raters will formally code the video recordings for analysis. This involves dividing up the coding so that each rater will be responsible for coding 1 of the 4 study sites (ie, 6 sessions per rater).

We will analyze all 6 video recordings from each data collection site. These comprise recordings of the first 2 sessions (1 and 2), the middle 2 sessions (11 and 12), and the final 2 sessions (19 and 20). Each rater will code the entirety of each selected session (60 minutes), with each bowling turn of each participant analyzed. Then, we will combine coded data from the 6 sessions into 3 respective time points: start (T1) = the first 2 sessions, midpoint (T2) = the 2 middle sessions, and end (T3) = the last 2 sessions.

## Results

The motion-based technology intervention has the potential to positively impact participants’ physical function, specifically balance (as measured through the Mini-BESTest) and movement confidence (analyzed from coded video recordings). This could confirm the feasibility and potential benefits of using motion-based technology to deliver exercise interventions to people with dementia or MCI. There is also potential for the motion-based technology intervention to positively impact the cognitive function of people with dementia or MCI (as measured through MoCA score), offering new approaches for cognitive rehabilitation with this population. The project was funded in 2019 and enrollment was completed on February 28, 2020. Data analysis is underway and the first results are expected to be submitted for publication in 2021.

## Discussion

### Principal Findings

This study is designed to examine the impacts of a group motion-based technology intervention on balance, movement confidence, and cognitive function among people with dementia or MCI. To our knowledge, this is the first study in an emerging body of literature to investigate these important outcomes with regard to the use of motion-based technology for people with dementia or MCI.

### Limitations

Despite the suggested contributions of the study, the proposed study features several potential limitations. First, there is a high risk of researcher bias given that the first author (ED) will be the one to conduct all pre- and posttest measures (ie, MoCA and Mini-BESTest), facilitate the study intervention with participants, and assist with analyzing participants’ movement confidence data. Thus, we recommend that future intervention studies of this nature be carried out with a larger research team where different members can be responsible for conducting different aspects of the study (ie, 1 person conducts the pre- and posttest measures, 1 person facilitates the intervention, and at least two separate individuals analyze the movement confidence data). However, from an opposite perspective, it could be considered positive that the first author (ED) was involved in all aspects of this preliminary study, in order to comment on feasibility and effectiveness, which can help to inform future studies of a similar nature.

Second, we will recruit participants via self-referral to a study advertisement, which suggests that participants who volunteer to take part in the study may be more keen to improve their balance, movement confidence, and cognitive function, which could influence their willingness to take part in the intervention.

Third, we expect that 40% of recruited participants will not complete the study intervention due to reasons outside of our control (eg, prolonged illness, moving to long-term care).

Fourth, while this study is adequately powered, the sample size is still considered small. However, small sample sizes are common in many rehabilitation studies due to lack of resources, lack of funding, recruitment challenges, and high dropout rates [53].

Fifth, the demographic questionnaire developed for this study does not capture participants’ specific diagnosis (eg, Alzheimer disease) or the number of years since their diagnosis. Indeed, it cannot be ruled out that these demographic variables could impact participants’ level of movement confidence, as well as their ability to improve from the intervention. 

Sixth, we acknowledge that the methods being used to evaluate movement confidence (ie, analysis of coded video recordings) are pilot in nature, meaning that there is a chance that some aspects of movement confidence may not be captured using the proposed approach. We recommend that future studies also include a self-report measure or qualitative methods such as interviews, or both, to capture participants’ true feeling of movement confidence.

Seventh, we will recruit a convenience sample of participants from 4 community-based adult day programs near Toronto, Canada. As such, all study participants will live in the community and come from 1 general geographical location. Thus, the findings of this study may not be generalizable to people living in different geographical locations (eg, rural and remote locations) or people living in different care settings (eg, long-term care homes). Eighth, we acknowledge that the game of bowling is not culturally inclusive and may be familiar only to people from certain cultural backgrounds (eg, North American). As a result, choosing the game of bowling may have the potential to impact the diversity of our study sample, as people who are not familiar with the game may be less likely to take part in the study.

### Comparison With Prior Work

The findings of the proposed study can contribute to the literature in several regards. First, the results of this study could provide further insight into the potential impacts of motion-based technology for people with dementia or MCI, which could stimulate further outcomes-based research in this area. Second, this work can be used to inform the development and design of motion-based technology games for people with dementia or MCI. To date, commercially available motion-based technology games have not been targeted toward people with dementia or MCI, which likely plays a role in the lack of rehabilitation literature regarding the impacts of motion-based technology interventions for this population [38]. Indeed, there is a need for further research using motion-based technology systems and games that are specifically designed to be enjoyable and accessible for this population [28]. For example, systems could be developed that provide in-game prompts, thus reducing the demands on an external (eg, human) facilitator. Similarly, errorless learning capabilities among people with dementia or MCI [54] could be leveraged within motion-based technology games by creating a wider repertoire of games that can be broken down into procedural steps.

Broadly, we expect that this research will be relevant to scientific, clinical, and professional audiences. For example, we expect that this research will be relevant to scholarly rehabilitation practice, advocacy, or advancing understanding of occupation (ie, meaningful activity) as a fundamental social determinant of health and well-being for people with dementia or MCI. Understanding the potential impacts of using motion-based technology with people with dementia or MCI can inform future evidence-based, community-based rehabilitation practice. That is, if participants show significant improvements in balance, movement confidence, and cognitive function as a result of partaking in the motion-based technology intervention, there is the potential of motion-based technology systems being incorporated into rehabilitation interventions targeting these outcomes. Additionally, we expect that this study will demonstrate the feasibility of using motion-based technology to deliver task-specific interventions to people with dementia or MCI. This is relevant given that tasks and goals of importance to clients are often used to inform rehabilitation interventions. Finally, this research aims to advocate the inclusion of people with dementia or MCI in rehabilitation science and interventions. We expect that this study will demonstrate the ability of people with dementia or MCI to engage in meaningful activity and emphasize the importance of meaningful activity to support health and well-being of people with dementia or MCI.

### Conclusions

The purpose of the proposed study is to examine the impacts of a group motion-based technology intervention on balance, movement confidence, and cognitive function among people with dementia or MCI. The findings of this study could confirm the feasibility and potential benefits of using motion-based technology to concurrently deliver cognitive and physical interventions to people with dementia or MCI. Confirming the feasibility of potential benefits of using motion-based technology with people with dementia or MCI could have several implications for research, clinical practice, and recreational care targeting this population.

## Abbreviations

MCI: mild cognitive impairment
Mini-BESTest: Mini-Balance Evaluation Systems Test
MoCA: Montreal Cognitive Assessment
